# Accuracy of a portable accelerometer-based navigation system for cup placement and intraoperative leg length measurement in total hip arthroplasty: a cross-sectional study

**DOI:** 10.1186/s12891-021-04167-y

**Published:** 2021-03-23

**Authors:** Hiromasa Tanino, Yasuhiro Nishida, Ryo Mitsutake, Hiroshi Ito

**Affiliations:** Department of Orthopaedic Surgery, Asahikawa Medical University, Midorigaoka-Higashi 2-1-1-1, Asahikawa, 078-8510 Japan

**Keywords:** Navigation, Portable, Cup position, Leg length, Total hip arthroplasty

## Abstract

**Background:**

Complications after total hip arthroplasty (THA) are frequently the consequence of malpositioned components or leg length discrepancy after surgery. Recently, a new version of a portable, accelerometer-based hip navigation system (New HipAlign) was made available with a change in the method of measuring cup abduction and the addition of a leg length measurement function. The purposes of this study were to investigate cup positioning and to examine the accuracy of leg length measurement with New HipAlign.

**Methods:**

Cups were implanted and intraoperative leg length change was measured using New HipAlign in 60 THAs through a posterior approach in the lateral decubitus position. The cup position and radiographic leg length change were determined postoperatively on pelvic radiograph and computed tomography scans. We previously compared cup positioning with a previous version of a portable, accelerometer-based hip navigation system (Previous HipAlign) and conventional surgical techniques. Cup positioning in this study was compared with the results of out previous study using Previous HipAlign.

**Results:**

The mean cup abduction of 40.3° ± 4.9° (range, 26° to 53°) and the mean cup anteversion of 15.8° ± 5.6° (range, 6.7° to 29.5°) were found. The deviation of the postoperative measured angles from the target cup position was 3.7° ± 3.3° for cup abduction and 5.9° ± 3.6° for cup anteversion. 56/60 of the cups were inside the Lewinnek safe zone. Compared with our previous study using Previous HipAlign, there were no significant differences with regard to cup abduction, cup anteversion, the deviation from the target cup position for cup abduction, the value of deviation for cup anteversion, and the number of cups inside the Lewinnek safe zone (*P* = 0.218, 0.334, 0.651, 0.797, 0.592). The mean difference between the intraoperative and radiographic leg length changes was + 0.8 ± 3.4 mm. There was significant correlation between the intraoperative and radiographic leg length changes (*r* = 0.804, *P* = 0.000).

**Conclusions:**

Use of New HipAlign allowed for accurate cup placement and reliable leg length measurement during THA.

**Trial registration:**

Clinical trial is defined as *‘any research study that prospectively assigns human participants or groups of humans to one or more health-related interventions to evaluate the effects on health outcome’ by* the World Health Organization (WHO)*.* Because this study is not a clinical trial, trial registration is not needed.

## Background

Complications after total hip arthroplasty (THA) are frequently the consequence of malpositioned components or leg length discrepancy (LLD) after surgery [1]. Achieving accurate cup positioning is important to prevent dislocation, impingement, liner fracture, and long-term wear [2]. Cup positioning using the conventional surgical technique, even in the hands of experienced surgeons leads to variations between the actual and desired cup position because patient positions on the table is variable [3].

Residual LLD after THA can result in gait disorders, knee and back pain, abnormal force transmission across the hip, early revision surgery, and patient dissatisfaction [4]. Although a number of methods have been described to measure leg length change during surgery, achieving equal leg lengths in primary THA still remains a surgical challenge in current orthopaedic surgery. Many studies have demonstrated that navigation system is associated with decreased variability in the mean cup abduction angle, decreased variability in the mean cup anteversion angle, and an increased likelihood of placement of the acetabular cup within the so-called safe zone [1, 5–11], and navigation system allows surgeons to evaluate leg length intraoperatively [1, 12–17].

Despite these advantages of navigation system, the widespread adoption of this technology has been limited. This is likely because a lack of evidence of clinical effect and concerns regarding increased operative time and cost [6, 7, 18]. Recently, a portable, accelerometer-based navigation system for THA (HipAlign; OrthAlign Inc., Aliso Viejo, CA, USA) was introduced, and improved cup positioning have been reported in several studies [19–23]. After our previous study [24], a new version of a portable, accelerometer-based hip navigation system (New HipAlign) was made available with a change in the method of measuring cup abduction and the addition of a leg length measurement function. To our knowledge, there is no study that reports the results of New HipAlign of the lateral decubitus position. Therefore, the purposes of this study were to: (1) investigate cup positioning with New HipAlign. (2) examine the accuracy of leg length measurement using HipAlign. We hypothesized that the accuracy of cup placement would be similar for New HipAlign and the previous version of portable, accelerometer-based hip navigation system (Previous HipAlign), and the accuracy of leg length measurement would be reliable.

## Methods

### Patients

This study analyzed 69 consecutive THAs performed with the use of New HipAlign between May 2019 to February 2020. This study was approved by the Institutional Review Board of Asahikawa Medical University (AMU19243). Patients with revision THA, prior hip surgery, or preoperative LLD greater than 2.5 cm were excluded [25]. Nine patients (9 hips) were excluded from this study: prior hip surgery (4 hips), preoperative LLD greater than 2.5 cm (3 hips), and navigation terminated intraoperatively (2 hips). The analyses were carried out on the remaining 60 procedures. The average age was 64 years (range, 24–87 years), and height and body weight averaged 156 cm and 62 kg, respectively. There were 45 women and 13 men, with 31 left and 29 right THAs. The preoperative diagnoses were osteoarthritis in 51, and osteonecrosis of the femoral head in 9. Two patients had bilateral THAs. Demographic and perioperative data are presented in Table 1.

**Table 1:** Group characteristics

	Previous version group (from our previous study [24])	New version group	*P* value
No. of hips/patients	55 hips (54)	60 hips (58)	
Gender, Male/Female	10/44	13/45	0.610
Age (years)	65.7 ± 11.0	64.3 ± 15.5	0.981
Height (cm)	154.3 ± 7.4	155.8 ± 10.8	0.834
Weight (kg)	58.6 ± 11.2	62.3 ± 14.8	0.325
Side affected, left/right	28/27	31/29	0.935
Type of femoral component, CMK/S-ROM	51/4	49/11	0.079
Preoperative diagnosis ^a^			0.725
OA	48	51	
ON	5	9	
RA	2	0	
Operation time (minutes)	79.8 ± 13.0	78.7 ± 12.0	0.564

*OA* Osteoarthritis, *ON* Osteonecrosis of the femoral head, *RA* Rheumatoid arthritis

Values are given as the mean and the standard deviation, except for number of hips/patients and preoperative diagnosis

No data missing

^a^ The preoperative diagnoses were categorized into 2 classes: 1) OA or 2) ON and RA for statistical analysis

### Surgical procedure

The surgery was performed at one center by two senior surgeons (HT and HI), the same as in our previous study [24]. A standard posterior approach in a lateral decubitus position was used in all cases. All patients received a THA using New HipAlign for cup placement and intraoperative leg length measurement. Each surgeon had used the navigation system for more than forty THAs, prior to this study.

**Fig. 1:**
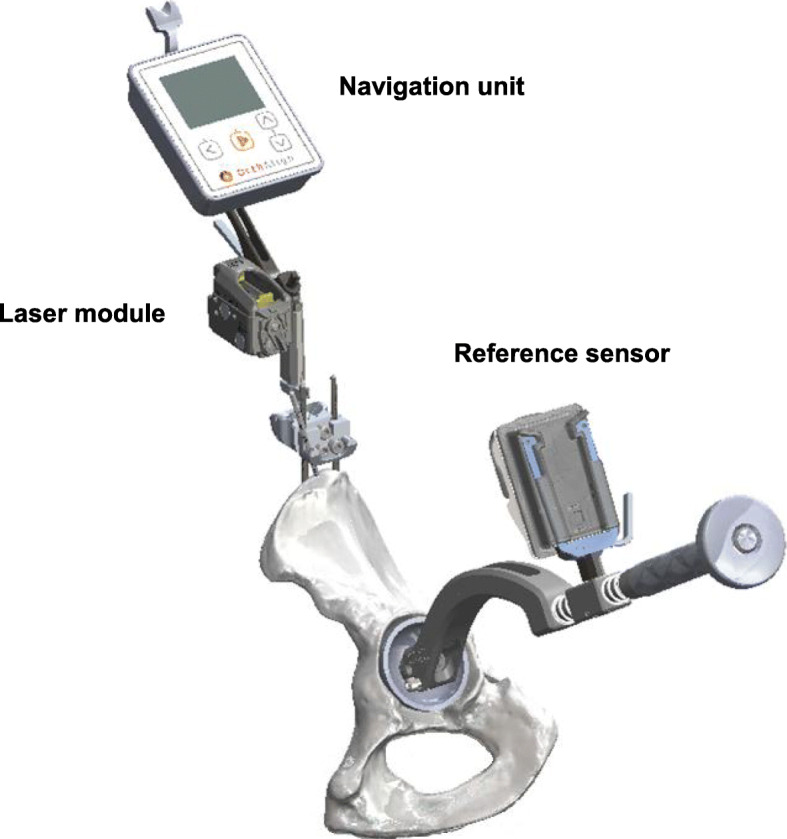
New HipAlign

**Fig. 2:**
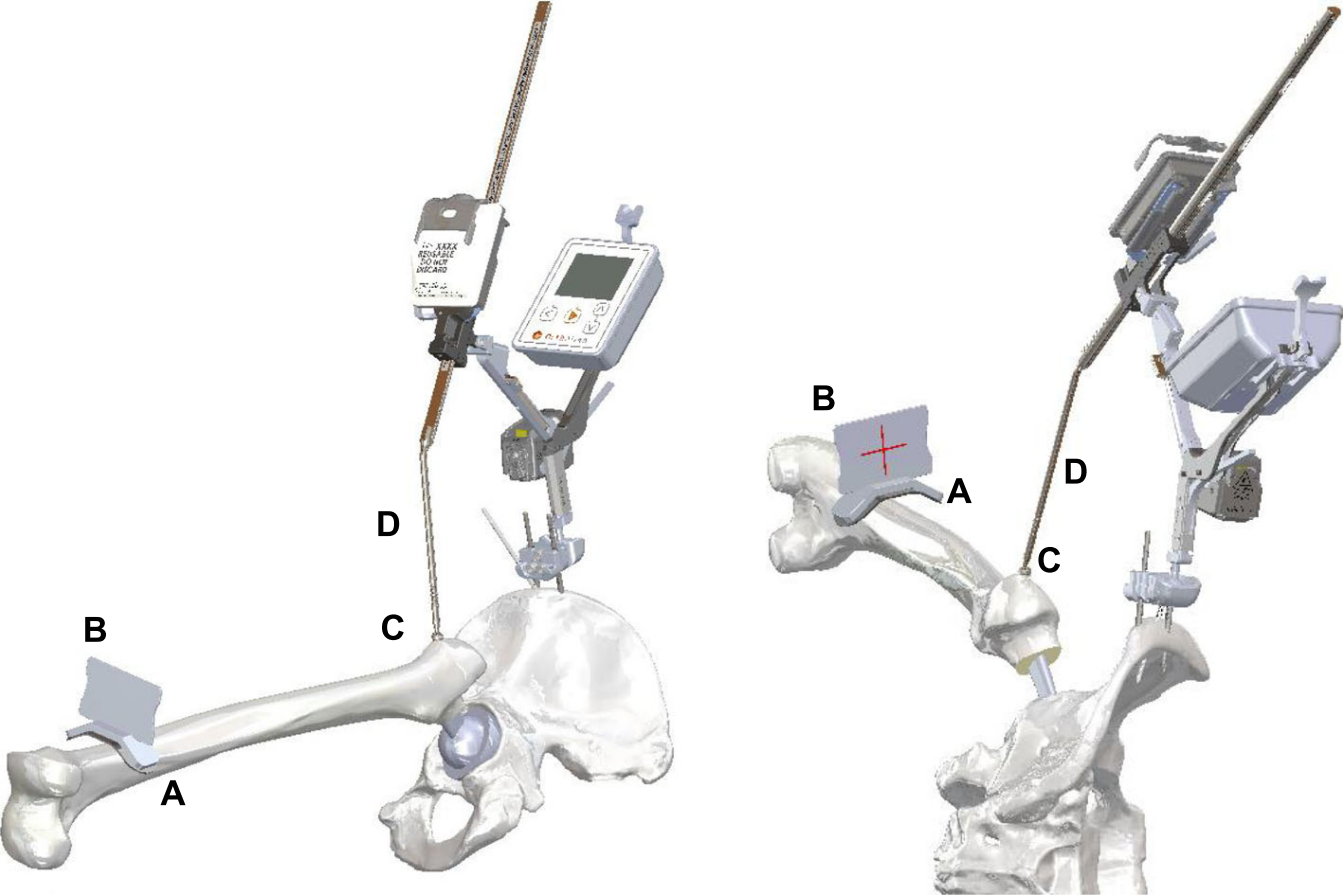
Intraoperative leg length measurement. The thigh plate (**a**) was positioned laterally on the distal thigh (not shown) and secured with an incision foil, not fixed to the femoral bone. After inserting the femoral implants and hip reduction, the limb was repositioned in the initial neutral reference position by the laser projection aligned with the marking on the vertical target (**b**), and the femur screw (**c**) was re-registered using the registration probe (**d**)

The HipAlign system consists of a disposable navigation unit, which houses the display console and a reference sensor (Fig. 1). The navigation unit and reference sensor each contain triaxial accelerometers and gyroscopes that communicate wirelessly with one another. The navigation unit and reference sensor were paired and calibrated on a flat table prior to surgery. The metal pelvic base and navigation unit were percutaneously secured with two parallel 3.2 mm pins and one oblique pin to the ipsilateral iliac crest using sterile techniques. Before incision, the longitudinal coronal plane of body was registered by holding the registration probe parallel to long axis of body. In Previous HipAlign, the angle between a line drawn through both acetabular teardrops and a line from the most lateral point of acetabular rim through the bottom of the acetabular teardrop (RT angle) was measured from pelvic anteroposterior radiograph before surgery, and input into the navigation unit as part of the setup. And four points around the acetabulum were registered using the registration probe before acetabular preparation. These steps are eliminated in New HipAlign. The system calculates the reference plane from coronal registration and gravity. For intraoperative leg length measurement, the laser module was attached to bracket of a navigation unit, and the thigh plate was positioned laterally on the distal thigh and secured with an incision foil. Prior to the femoral head dislocation and neck osteotomy, a small reference screw was put into the greater trochanter for femoral registration, and the vertical target was magnetically attached to the thigh plate. A neutral reference position of the limb was defined as 0° of flexion, abduction, and internal/external rotation. The limb was positioned in the neutral reference position, and the laser projection on the vertical target was traced with a marking pen. The femur screw was registered using the registration probe and was used to measure leg length changes. During placement of the final acetabular component, the reference sensor was attached to the cup impactor. Radiographic cup abduction and anteversion were displayed on the display console [26, 27]. Our target cup position for all patients was 40° abduction and 20° anteversion, similar to Domb et al. [10]. Supplemental screw fixation was used in all hips. Following screw fixation, cross-linked polyethylene was inserted. After inserting the trial or final femoral implants and hip reduction, the limb was repositioned in the initial neutral reference position by the laser projection aligned with the marking on the vertical target, and the femur screw was re-registered using the registration probe (Fig. 2). Our previous study showed that the range of internal rotation with 90° hip flexion and 0° abduction/adduction was a useful method to predict postoperative stability after THA [28]. The target angle of stem anteversion was decided by the intraoperative stability test, and the stem was placed at the target angle using the angle-measuring instrument [29]. The navigation system calculates and displays intraoperative leg length change which is the difference between the length of the operative leg before the femoral head dislocation and the length of the operative leg after inserting the final femoral implant and hip reduction. The surgeon aimed to restore the leg length according to their preoperative plan. Leg length was finally adjusted by changing the neck length of the femoral head. All patients underwent THA using cementless, hemispherical acetabular component (Continuum, Zimmer, Warsaw, IN, USA) and 32 mm ceramic head. Most of femoral stems used in this study were cemented CMK Original Concept stem (Zimmer, Warsaw, IN, USA), but modular cementless stems (S-ROM; Depuy, Warsaw, IN, USA) were used in eleven hips. All patients were allowed full weight-bearing in the immediate postoperative period, and they were encouraged and assisted to commence walking.

### Data collection

Pelvic anteroposterior radiographs were obtained in the supine position before surgery and 1 week after surgery, and were used to measure cup abduction and for leg length measurements. Cup abduction was measured as the angle between a line drawn through both acetabular teardrops and a line through the face of the acetabular component. Leg length was obtained by drawing a line through both acetabular teardrops and measuring the distance to the superior margin of the lesser trochanter. Radiographic leg length change was obtained by comparing preoperative and postoperative radiographs. Measurements were calibrated to a radiopaque standardized metal sphere or the known size of the prosthetic head to assess the degree of magnification. The difference between the intraoperative and radiographic leg length changes was defined as the accuracy of leg length measurement. Positive value of the accuracy of leg length measurement was recorded when the radiographic leg length change was longer, and negative value indicated the radiographic leg length change was shorter than the intraoperative leg length change. And the absolute positive value of the accuracy of leg length measurement was defined as the absolute accuracy of leg length measurement. A pelvic computed tomography (CT) scan was performed 2 weeks after surgery. Scans were acquired in the supine position. The cup anteversion was measured on the broadest axial cut of the cup by the angle the cup margins subtended on the perpendicular drawn to the horizontal tangent along the ischia. These postoperative angles were digitally measured (ViewR, YOKOGAWA, Japan) by one observer, the same as in our previous study [24]. And radiographic leg length changes were digitally measured (ViewR) by same observer. For all analyses, the radiographic angle values were used based on the definitions by Murray [27]. To account for intraobserver and interobserver reliability, the author measured all radiographs and CT scans twice and they were also measured by another author independently. The duration of each operation was recorded. The main outcome measures were postoperative cup abduction and anteversion angles, the absolute deviation of the postoperative measured angle from the target position, the number of cups inside the Lewinnek safe zone (30° – 50° of abduction and 5° – 25° of anteversion) [2], operation time, and the accuracy of leg length measurement. The cup position and operation time in this study using New HipAlign (new version group) were compared with those of 54 patients (55 hips), who underwent a THA using Previous HipAlign in our previous study (previous version group) [24] (Table 1).

### Statistical analysis

In our previous study [24], a sample size of 55 hips was required for previous version group. In this study, it was planned to include more than 55 hips in the new version group to compare the results of the previous version group.

For continuous variables, the normality of the data was assessed using a Shapiro-Wilk test, and statistical analysis was done with a two-tailed independent t-test or a nonparametric Mann-Whitney U test. Categorical data were analyzed using the chi-square test. Differences in variation between groups were assessed using a Levene test. The Pearson correlation coefficient was used to compare intraoperative and radiographic leg length changes. Correlation coefficients > 0.8 were judged as good, and hence as confirming a substantial reliability of the measurement method. The intraobserver reliability and interobserver reliability were evaluated by using the intraclass correlation coefficient (ICC). A *P* < 0.05 was considered significant. Statistical analyses were performed using SPSS Version 24 (SPSS Inc., Chicago, IL, USA).

## Results

There were no significant differences with regard to gender, age, height, body weight, side, type of femoral component, and preoperative diagnosis between the new version group and previous version group (Table 1).

**Fig. 3:**
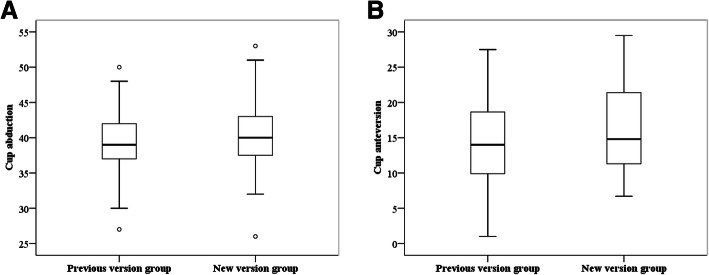
Box plots of postoperative abduction (**a**) and anteversion (**b**) angles of the acetabular component in the previous version group and new version group. The mean postoperative cup abduction of the acetabular component was 39.2° ± 4.6° (range, 27° to 50°) in the previous version group and 40.3° ± 4.9° (range, 26° to 53°) in the new version group. The mean postoperative cup anteversion was 14.6° ± 6.1° (range, 1° to 27.5°) in the previous version group and 15.8° ± 5.6° (range, 6.7° to 29.5°) in the new version group. There were no significant differences between the previous version group and new version group with regard to mean cup abduction and anteversion (*P* = 0.218, 0.334). Significant differences in variation between groups were not indicated for cup abduction and anteversion (*P* = 0.767, 0.870). Results of previous version group were from our previous study [24]

The mean postoperative cup abduction of the acetabular component was 40.3° ± 4.9° (range, 26° to 53°) in the new version group and 39.2° ± 4.6° (range, 27° to 50°) in the previous version group. The mean postoperative cup anteversion of the acetabular component was 15.8° ± 5.6° (range, 6.7° to 29.5°) in the new version group and 14.6° ± 6.1° (range, 1° to 27.5°) in the previous version group. There were no significant differences between the new version group and previous version group with regard to the mean cup abduction and anteversion (*P* = 0.218, 0.334) (Fig. 3a and b).

The absolute deviation of the postoperative measured angles from the target position for the cup abduction was 3.7° ± 3.3° (range, 0° to 14.0°), and the value of deviation for the cup anteversion was 5.9° ± 3.6° (range. 0.2° to 13.3°). There were no significant differences between the new version group and previous version group with regard to the absolute deviation of the postoperative measured angles from the target position for the cup abduction and the cup anteversion (*P* = 0.651, 0.797) (Table 2).

**Table 2:** Absolute deviation of the postoperative measured angles from the target position

	Previous version group (from our previous study [24])	New version group	*P* value
Cup abduction	3.7° ± 3.0°	3.7° ± 3.3°	0.651
Cup anteversion	6.0° ± 4.5°	5.9° ± 3.6°	0.797

No data missing

**Fig. 4:**
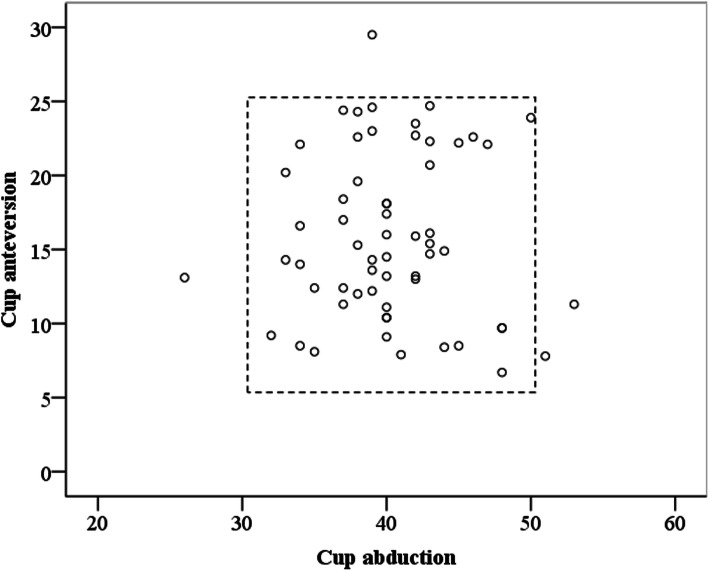
Cup positions relative to Lewinnek safe zone [2] in the new version group. The number of cups inside the Lewinnek safe zone was 51/55 (93%) and 56/60 (93%) in the previous version group and new version group (*P* = 0.592). Results of previous version group were from our previous study [24]

The number of cups inside the Lewinnek safe zone was 56 (93%). There was no significant difference between the new version group and previous version group in the rate of cups inside the Lewinnek safe zone (*P* = 0.592) (Fig. 4).

**Fig. 5:**
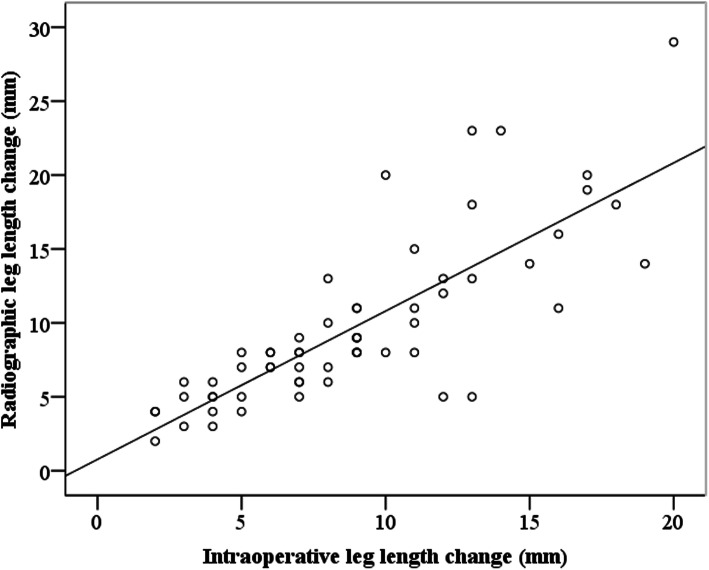
Correlation between the intraoperative and radiographic leg length changes

The mean intraoperative leg length change was + 8.9 ± 4.5 mm (range: + 2.0 to + 20.0 mm). The mean radiographic leg length change was + 9.7 ± 5.6 mm (range: + 2.0 to + 29.0 mm). The mean accuracy of leg length measurement was + 0.8 ± 3.4 mm (range, − 8 to 10 mm). The mean absolute accuracy of leg length measurement was 2.3 ± 2.6 mm (range, 0 to 10 mm). There was significant correlation between the intraoperative and radiographic leg length changes (*r* = 0.804, *P* = 0.000) (Fig. 5).

The mean total operation time for the navigation-assisted implantation, including pin placement was 79 min (range, 60 to 108) and was similar to the previous version group (*P* = 0.564) (Table 1). We did not experience any interference between the screw inserted in the greater trochanter and stem during preparation of the femoral cavity. There were no complications arising at the pin sites, but one dislocation occurred within 3 months after surgery.

The measurements had an excellent ICC for intraobserver (0.96 cup abduction, 0.97 cup anteversion, 0.96 leg length change) and interobserver (0.97 cup abduction, 0.96 cup anteversion, 0.94 leg length change) correlation.

## Discussion

The purposes of this study were to evaluate the accuracy of New HipAlign for acetabular cup placement in THA, and to investigate the accuracy of leg length measurement of HipAlign. We hypothesized that the accuracy of cup placement would be similar for New HipAlign and Previous HipAlign, and the accuracy of leg length measurement would be reliable. This study demonstrates that the cups were placed accurately in the new version group, similar to the previous version group. Although the function of leg length measurement was added, mean operation time in the new version group was similar to the previous version group. This study also demonstrates that leg length measurement of HipAlign was reliable during THA.

Component malpositioning and postoperative LLD are the most common technical problems associated with THA [1]. Thus, control of implant positioning and leg length change during the surgery is crucial. Conventional techniques to determine cup position intraoperatively historically have consisted of freehand techniques and the use of mechanical guides. There are multiple studies which show that surgeons using conventional technique often place the cups outside the safe zone and there is significant variability in final cup position [30].

Several meta-analyses showed that navigation system decreased the number of acetabular cups implanted outside the safe zone. Gandhi et al. and Moskal et al. reported the rates of cups inside the safe zone in the navigation group were 89.3 and 80.75% in their meta-analyses [31, 32]. The rates of cups inside the safe zone in the new version group and previous version group were similar or slightly higher than the rates reported by their meta-analyses.

Hasegawa et al. stated, in CT-based navigation, the absolute values of abduction errors were reported to range from 1.2° to 4.6°, and the anteversion errors ranged from 1.0° to 4.4°. In imageless large-console navigation, the abduction errors were reported to range from 2.9° to 3.2°, and the anteversion errors ranged from 3.7° to 6.5°. The values of deviation for cup abduction and anteversion in the new version group and previous version group were similar or slightly larger than the values reported by Hasegawa et al. [23]. Cup abduction was measured based on registration of RT angle and gravity with Previous HipAlign. With New HipAlign, several steps for measuring cup position are eliminated, and cup abduction was measured based on gravity at the time of registration. The results in this study showed the accuracy of cup placement was similar in the new version group and previous version group.

Various methods have been described to measure leg length change during surgery with varying results. These include the shuck test, comparing the dimensions of the resected bone with the dimensions replaced by the prosthesis, comparison of surgery with the detailed preoperative surgical plan, the use of mechanical jigs and measuring calipers, and the use of navigation systems [1, 15, 16, 33–36]. Many studies did not discuss the accuracy of measurement technique or report the correlation between the predicted and the actual leg length changes [36, 37].

Our results in this study were compared with previous studies (Table 3). Correlation between intraoperative and radiographic leg length changes was fair [38, 39] or good [36, 40] with caliper, and was good with the navigation system [13, 14, 17, 40] also in this study. And the accuracy of leg length measurement in this study was equivalent to large console navigation systems [1, 8, 12–14, 17, 40].

**Table 3:** The accuracies of the leg length measurements in the present and previous studies

	Method	Correlation between intraoperative and radiographic leg length changes. The Pearson correlation coefficient and *P* value	Accuracy of leg length measurement (mm)
Ranawat [36]	Caliper	0.82, significant*	–
Rice [38]	Caliper	0.275, *P* > 0.05	3.2 ± 8.0 (absolute value)
Enke [39]	Caliper	0.259, not significant	–
Renkawitz [13, 14, 17]	Imageless navigation (cadaver study)	0.83, *P* < 0.001*	0.74^a^
	Imageless navigation (cadaver study)	0.92, *P* < 0.001*	0.50^a^
	Imageless navigation	0.80, *P* < 0.001*	0.35^a^
Kitada [8]	CT-based navigation	–	1.3 ± 4.1^a^
Ogawa [40]	CT-based navigation	0.88, *P* < 0.001*	2.4 ± 1.7 (absolute value)
	Caliper	0.89, *P* < 0.001*	2.1 ± 1.6 (absolute value)
Ecker, Murphy and Ecker [1, 12]	CT-based navigation	–	−0.5 ± 1.7^a^
This study	Portable navigation	0.80, *P* = 0.000*	0.8 ± 3.4^a^
			2.3 ± 2.6 (absolute value)

*Statistically significant

^a^Values are not absolute values

HipAlign, a recently introduced, portable, accelerometer-based navigation system for THA, has been developed in the attempt to achieve the same precision of cup placement as that with the large console navigation system, with the ease of use and convenience of conventional surgical techniques. The function of leg length measurement was added in New HipAlign. The leg length measurement function of HipAlign could be advantageous for the surgeons, who could use this new function to know how to adjust the leg length intraoperatively. HipAlign does not require the capital expense that is required for navigation or robotic systems. HipAlign only requires the per case cost of the disposable unit ($1100) [24]. In our previous study, HipAlign added 10 min per procedure compared to the conventional technique; however, it does not add as much time as alternative technologies [24]. Recently, several studies reported the variation between the actual and desired cup position, and/or the difference between the navigation recorded and actual cup position [20, 22, 23]. Hasegawa et al. compared six studies of HipAlign for the absolute values of errors and reported HipAlign of the supine position seemed to be better for cup anteversion accuracy [23]. When HipAlign is used in the supine position, the functional pelvic plane is used for coronal registration, and the plane is set by sensing the direction of gravitational acceleration with a navigation sensor. When used in the lateral decubitus position, the longitudinal coronal plane of body is registered by holding the registration probe parallel to long axis of body. The accuracy of cup placement with HipAlign of the supine position and HipAlign of the lateral decubitus position might be different due to the difference in coronal registration. However, only our previous study [24] was included as the study using HipAlign of the lateral decubitus position in their study [23]. Surgeons need more data of HipAlign, especially for HipAlign of the lateral decubitus position. New HipAlign is a current version of HipAlign, and this study is the first study to investigate the results of cup positioning with New HipAlign including the results of leg length measurement with HipAlign of the lateral decubitus position. Navigation system allows the surgeon to assess the acetabular component position in reference to anatomic landmarks in real time, and many studies have confirmed that navigation system results in fewer malpositioned components [1, 5–11]. However, there is little evidence navigation system impacts clinical outcomes such as the rates of dislocation or revision surgery. Recently, several studies reported short-term clinical results of navigation THA using registry data. Aoude et al. reported navigation system reduced minor adverse events [41]. Montgomery et al. found no difference in dislocation rate between navigation THA and conventional THA and identified a higher early revision rate [42]. Bohl et al. showed decreased dislocation and aseptic acetabular revision rates who underwent THA with navigation system [43]. Gausden et al. reported the use of navigation system resulted in lower arthroplasty-related complications and readmission rates compared with conventional THA [44]. It is difficult to determine at this time whether innovations in navigation or robotic assistance really improve clinical results [45]. Future studies are requested to determine the benefit-cost ratio of a new technology.

There are several limitations in this study. The first limitation is the pelvic tilt. The surgery was performed in a lateral decubitus position, but postoperative pelvic radiographs and CT were obtained in the supine position. Zhu et al. described the pelvis tilted a mean of approximately 4° to 5° as patients move from the supine to the lateral position, supine to standing position, and between the preoperative and postoperative supine position [26]. The effect of pelvic tilt was not considered in this study. Although three-dimensional planning software using CT data enables to measure cup position after adjusting pelvic tilt, obliquity, and axial rotation, measuring methods of cup position in this study were same as those in our previous study [24]. A future study should consider this effect. The second limitation is this study does not present clinical outcomes data. Future studies must assess the effects of cup positioning and leg length measurements on clinical outcomes. The third limitation is the safe zone. Because the Lewinnek safe zone has been commonly referenced in studies, this study used the Lewinnek safe zone as reference. However, several studies have questioned whether the historic concept of a safe zone is clinically useful and redefined the safe zone [30, 46]. And a recent study described the importance of functional cup position [45]. It is possible for HipAlign to use different target cup position and different safe zone as reference. The fourth limitation is radiographic leg length measurements. Measurements from plain radiographs are susceptible to errors since horizontal dimensional parameters are influenced by variations in positions of the pelvis relative to the plane of the film and the divergence of the X-ray beams. The reliability of the measurements is further reduced by the influence of pelvic tilt and rotation. We used the acetabular teardrop as the anatomical landmark for leg length measurements as this landmark has been found to be reliable and constant due to its vertical and rotational stability in association with different pelvic positions [47]. And the method of measuring leg length as the distance between a line drawn through both acetabular teardrops to the superior margin of the lesser trochanter has been used by many studies [1, 12, 17, 35–37, 39, 40]. To improve accuracy, patients were placed in a standardized position and we used magnification marker and digital software for our radiographic analysis.

## Conclusions

New HipAlign offers accurate cup placement, similar to Previous HipAlign. Intraoperative assessment of leg length change correlates positively with radiographic leg length change, and was reliable during THA.

However, future studies are required to determine the long-term advantages of HipAlign.

## Data Availability

The datasets used and/or analysed during the current study are available from the corresponding author on reasonable request.

## Abbreviations

THA: Total hip arthroplasty
LLD: Leg length discrepancy
CT: Computed tomography
ICC: Intraclass correlation coefficient
